# Double End Excavators

**Published:** 1858-04

**Authors:** 


### THE DOUBLE ENDED EXCAVATOR.

Is an instrument which should have a place in every Dentists case.

It is a right and left curved hoe, cutting *to* and *from* you, each instrument being the *pair*. I use about four sizes—from the most delicate necessary, to excavate the smallest approximate cavity in the front teeth to that size, strong enough for any case.

The angle of the instrument should not be flattened up to the shaft; but beveled rather short, so that the whole of the strength of the neck, may be had in the direction of the *force* in the line of the handle. The cutting edge should be oval and not square, so that the groove that it may be necessary to cut many times, may not be sharp and angular, but rounded and smooth, so that the cavity can be the most perfectly fitted in all its parts, and this principle holds good in regard to other excavators; all hatchets, hoes, drills, etc., ought to be made oval on the face, for reasons obvious to every good operator, if his attention be drawn to it. All cavities should be made smooth and free from irregularities, so that it may be the most perfectly filled. —— HOY.

We manufacture the above at $3 per dozen.

JNO. T. TOLAND.

